# Postprandial triglyceride levels affecting postprandial thyroid stimulating hormone levels may be responsible for the increased postprandial thyroid stimulating hormone levels in people with reduced lipid tolerance

**DOI:** 10.3389/fendo.2025.1522928

**Published:** 2025-02-13

**Authors:** Peipei Tian, Shaojing Zeng, Yilin Hou, Dandan Liu, Yamin Lu, Guangyao Song

**Affiliations:** ^1^ Department of Internal Medicine, Hebei Medical University, Shijiazhuang, Hebei, China; ^2^ Hebei Key Laboratory of Metabolic Diseases, Hebei General Hospital, Shijiazhuang, Hebei, China; ^3^ Department of Endocrinology and Metabolism, Cangzhou Central Hospital, Cangzhou, Hebei, China; ^4^ Department of Endocrinology, Hebei General Hospital, Shijiazhuang, Hebei, China; ^5^ Department of Endocrinology, Baoding First Central Hospital, Baoding, Hebei, China; ^6^ Department of Nuclear Medicine, Hebei General Hospital, Shijiazhuang, Hebei, China

**Keywords:** triglyceride, thyroid stimulating hormone, high-fat meal, oral fat tolerance test, normal fat tolerance, impaired fat tolerance

## Abstract

**Objective:**

In this study, we aimed to explore the relationship between postprandial triglyceride (TG) and postprandial thyroid stimulating hormone (TSH) levels and compare the postprandial TSH levels in participants with normal lipid tolerance and reduced lipid tolerance.

**Methods:**

A total of 81 eligible participants were enrolled and given a high-fat meal of 1500 kcal, and blood samples were collected at 2, 4, 6, and 8 hours. Fasting blood glucose, total cholesterol, high-density lipoprotein cholesterol, low-density lipoprotein cholesterol, and fasting and postprandial TG, triiodothyronine (T3), tetraiodothyronine (T4), and TSH levels were tested. Based on the postprandial serum TG level, participants were divided into the normal lipid tolerance group (NFT) and the decreased lipid tolerance group (IFT).

**Results:**

Postprandial TG levels increased in both the NFT and IFT groups and then decreased over time. A higher and delayed peak of postprandial TG was observed in the IFT group, and there were statistically significant differences in TG levels at each time point in both groups. The area under the curve (TGAUC) was an independent influencing factor for the area under the curve (TSHAUC) of TSH. Postprandial TSH levels in both groups reached a trough at 2 h and peaked at 6 h, with a higher peak in the IFT group. Except for 2 h, TSH levels were significantly different at all other time points. There was no statistically significant difference in T3 or T4 levels between the two groups, with opposite trends for TSH.

**Conclusion:**

After a high-fat meal is consumed, the postprandial TSH level is influenced by the postprandial TG level, which may be the reason for the decreased thyroid function in the population with reduced lipid tolerance.

**Clinical Trial Registration:**

http://www.chictr.org.cn/index.aspx, identifier ChiCTR1800019514.

## Introduction

Hypothyroidism is a common pathological state associated with thyroid hormone deficiency and includes subclinical hypothyroidism (SCH) and overt hypothyroidism. SCH is defined as elevated serum thyroid-stimulating hormone (TSH) and free thyroxine (FT4) levels within the lower limit of the normal range. Numerous studies have indicated that SCH is an independent risk factor for cardiovascular diseases (1–6). Some studies have indicated (7–9) that thyroid hormones are associated with various metabolic abnormalities, even at the lower end of the normal range, suggesting that it is crucial to identify the potential factors affecting thyroid function early. Although autoimmune diseases are commonly recognized causes of thyroid dysfunction, the risk factors that trigger hypothyroidism are not well understood. Therefore, further research on the etiology of SCH is warranted. Jankovic et al. (10) observed that obese patients with hypertriglyceridemia exhibit elevated TSH levels, which significantly decrease after serum triglyceride (TG) levels decrease following bariatric surgery. A previous study (11) showed a positive correlation between hyperlipidemia and the risk of SCH. In a prospective study, an exploratory analysis of the relationship between the components of metabolic syndrome and decreased thyroid function in participants with and without metabolic syndrome showed that metabolic syndrome increased the risk of developing SCH. An analysis of the individual components of metabolic syndrome revealed that high TG levels were associated with an increased risk of SCH (12). These studies suggest that thyroid function in patients with hypertriglyceridemia may be adversely affected by lipotoxicity. In populations with abnormal lipid metabolism, lipids have long-term effects, and exploring the relationship between abnormal lipid metabolism and the risk of developing SCH is crucial for the prevention and effective management of SCH. In recent years, many studies have shown that postprandial TG levels are closely associated with type 2 diabetes and cardiovascular disease (13–15). An increasing number of researchers are focusing on the importance of postprandial TG. However, previous studies have been based on the effect of fasting TG levels on thyroid function. Research has also indicated that, in normal individuals, TSH levels decrease 2 hours after a meal (16–18). It is unclear whether postprandial serum TG levels are related to postprandial serum TSH levels and whether postprandial TSH levels differ in different lipid tolerance populations. Therefore, this study aimed to observe the relationship between postprandial TG levels and postprandial TSH levels by performing a lipid tolerance test in volunteers with normal fasting TG and thyroid hormone levels. This study also aimed to compare postprandial TSH levels between participants with normal lipid tolerance and those with reduced lipid tolerance.

## Materials and methods

### Study sample

This study included volunteers aged 25–70 years from the endocrinology outpatient department of Hebei General Hospital from May 2018 to December 2019. This study was approved by the Ethics Committee of Hebei General Hospital (Approval No.: 2018 No. 2, Date: February 26, 2018) and registered with the Chinese Clinical Trial Registration Center (Registration No.: ChiCTR1800019514). All volunteers signed informed consent forms and completed questionnaires as required.

### Exclusion criteria

Vegetarians; individuals with hyperthyroidism and hypothyroidism, diabetes, heart disease, kidney disease, malignant tumours, acute or chronic blood diseases, and infectious diseases; those with a family history of endocrine-related diseases, such as familial hypercholesterolaemia; individuals currently taking medications that affect glucose and lipid metabolism or inflammation (fish oil, contraceptives, hormones, beta-blockers, diuretics, hypoglycaemic drugs, and lipid-lowering drugs); and those who had experienced stroke, pregnancy, mental disorders, surgery, trauma, or weight changes >3 kg within the past 3 months.

### Oral fat tolerance test

All participants were instructed to follow a normal diet for 1 week and abstain from foods high in fat and protein (a list of foods to avoid was provided to all participants 1 week before the test). The participants began fasting at 22:00 on the day before the oral fat tolerance test (OFTT) and continued until 08:00. Participants were asked to consume a high-fat meal within 10 minutes, and during the 8-hour test period, they were allowed to drink water freely but were prohibited from smoking, eating, or engaging in strenuous exercise. Blood samples were collected before the high-fat meal and 2, 4, 6, and 8 hours after the meal. The high-fat meal had a total caloric content of 1500 kcal, with fat accounting for 60% (900 kcal) (monounsaturated fatty acids, polyunsaturated fatty acids: saturated fatty acids ratio = 2:2:1), carbohydrates for 20% (300 kcal), and protein for 20% (300 kcal). The production of the high-fat meal was completed by the nutrition department of our hospital.

### Laboratory assays

Body mass index (BMI), systolic blood pressure(SBP), and diastolic blood pressure(DBP) of the participants were uniformly measured by the same physician. The serum levels of fasting blood glucose (FBG), TG, total cholesterol (TC), high-density lipoprotein cholesterol (HDL-C), low-density lipoprotein cholesterol (LDL-C), triiodothyronine (T3), thyroxine (T4), TSH and the postprandial serum levels of TG, T3, T4 after a high-fat meal were measured by a fully automated biochemical analyser.

### Definitions of clinical conditions

Definition of normal fasting TG level: According to the “Guidelines for the Prevention and Treatment of Dyslipidemia in Chinese Adults (Revised 2016)”, a fasting TG level<1.7 mmol/L is defined as normal fasting triglycerides (19).

Definition of postprandial TG elevation: Based on the consensus recommendations of the 2011 Greek Conference, a TG level >2.5 mmol/L 4 hours after a high-fat meal load was defined as postprandial hypertriglyceridaemia (20).

### Grouping of subjects

Participants were divided into two groups: normal fat tolerance group (NFT): (Fasting TG <1.7 mmol/L, postprandial 4-hour TG ≤2.5 mmol/L) and impaired fat tolerance group (IFT): (Fasting TG <1.7 mmol/L, postprandial 4-hour TG >2.5 mmol/L).

### Statistical analysis

Statistical analysis was performed using SPSS 25.0 software. Normally distributed continuous data were expressed as mean ± standard deviation, and non-normally distributed continuous data were expressed as median (interquartile range). The trapezoidal rule was used to calculate the AUC. The change in TG (△TG) was defined as the difference between the postprandial 4-hour value and the fasting value, and the change in TSH (△TSH) was defined as the difference between the postprandial 6-hour value and the fasting value. Two-way multilevel repeated-measures analysis of variance was used to analyse the data between groups. One-way repeated-measures analysis of variance was used to analyse the data within each group. Between-group comparisons were performed using the t-test for normally distributed data with equal variance; otherwise, non-parametric tests were used. Pearson’s correlation analysis was used to assess the strength of the association between normally distributed variables; otherwise, Spearman’s correlation analysis was used. Statistical significance was set at P < 0.05. Multiple linear regression analysis of the relationship between TGAUC and TSHAUC. We used PASS 2021 (v21.0.3) software to test the sample size and analysis a power.

## Results

### Comparison of baseline data between two groups

A total of 81 volunteers met the inclusion criteria. There were 45 participants (21 men and 24 women) in the NFT group and 36 (14 men and 22 women) in the IFT group. We used PASS 2021 (v21.0.3) software with a significance level of α =0.05. The sample size of the NFT group was 45, and that of the IFT group was 36 (4 h, 6 h, and 8 h). The minimum difference between the values was input into the software for calculation. The results showed that the sample size of this study achieved a power of 91.40%, which is higher than the required 90%. Therefore, our sample size was sufficient.

BMI, SBP, DBP, FBG, TG, 4-hour postprandial triglycerides (4hTG), HDL-C and TSH levels were significant differences in two groups (P < 0.05). We did not observe any significant differences in age, sex, TC, LDL-C, T3, T4, triiodothyronine area under the curve (T3AUC), and thyroxine area under the curve (T4AUC) between the two groups (P > 0.05) (**Table 1**).

**Table 1:** Comparison of baseline data between two groups.

	Total (n=81)	NFT (n=45)	IFT (n=36)
Age (year)	38.00 (27.50,53.50)	32.00 (28.00,51.00)	45.50 (27.00,56.25)
Male,n (%)	35 (43.2%)	21 (46.7%)	14 (38.9%)
BMI (kg/m^2^)	25.89 ± 3.77	23.61 ± 3.24	25.88 ± 3.77 *
SBP (mmHg)	126.31 ± 15.98	116.36 ± 14.34	126.31 ± 15.98 *
DBP (mmHg)	77.17 ± 8.59	73.11 ± 8.83	77.17 ± 8.59 *
FBG (mmol/L)	5.58 ± 0.85	5.16 ± 0.51	5.58 ± 0.85 *
TC (mmol/L)	4.63 ± 0.97	4.46 ± 0.91	4.63 ± 0.97
TG (mmol/L)	1.30 ± 0.25	0.90 ± 0.25	1.30 ± 0.25 **
4hTG (mmol/L)	3.34 ± 0.66	1.59 ± 0.45	3.34 ± 0.66 **
HDL-C (mmol/L)	1.18 ± 0.27	1.32 ± 0.27	1.18 ± 0.27 *
LDL-C (mmol/L)	2.99 ± 0.66	2.74 ± 0.69	2.99 ± 0.66
T3 (nmol/L)	1.64 ± 0.30	1.61 ± 0.23	1.64 ± 0.30
T4 (nmol/L)	89.09 ± 20.20	91.35 ± 16.00	89.09 ± 20.20
TSH (uIU/mL)	1.82 (1.38,2.34)	1.52 (0.99,2.02)	1.82 (1.38,2.34) *

*P<0.05, compared with NFT group. **P<0.001, compared with NFT group.

### Comparison of the Serum TG levels between two groups during the OFTT

**Figure 1:**
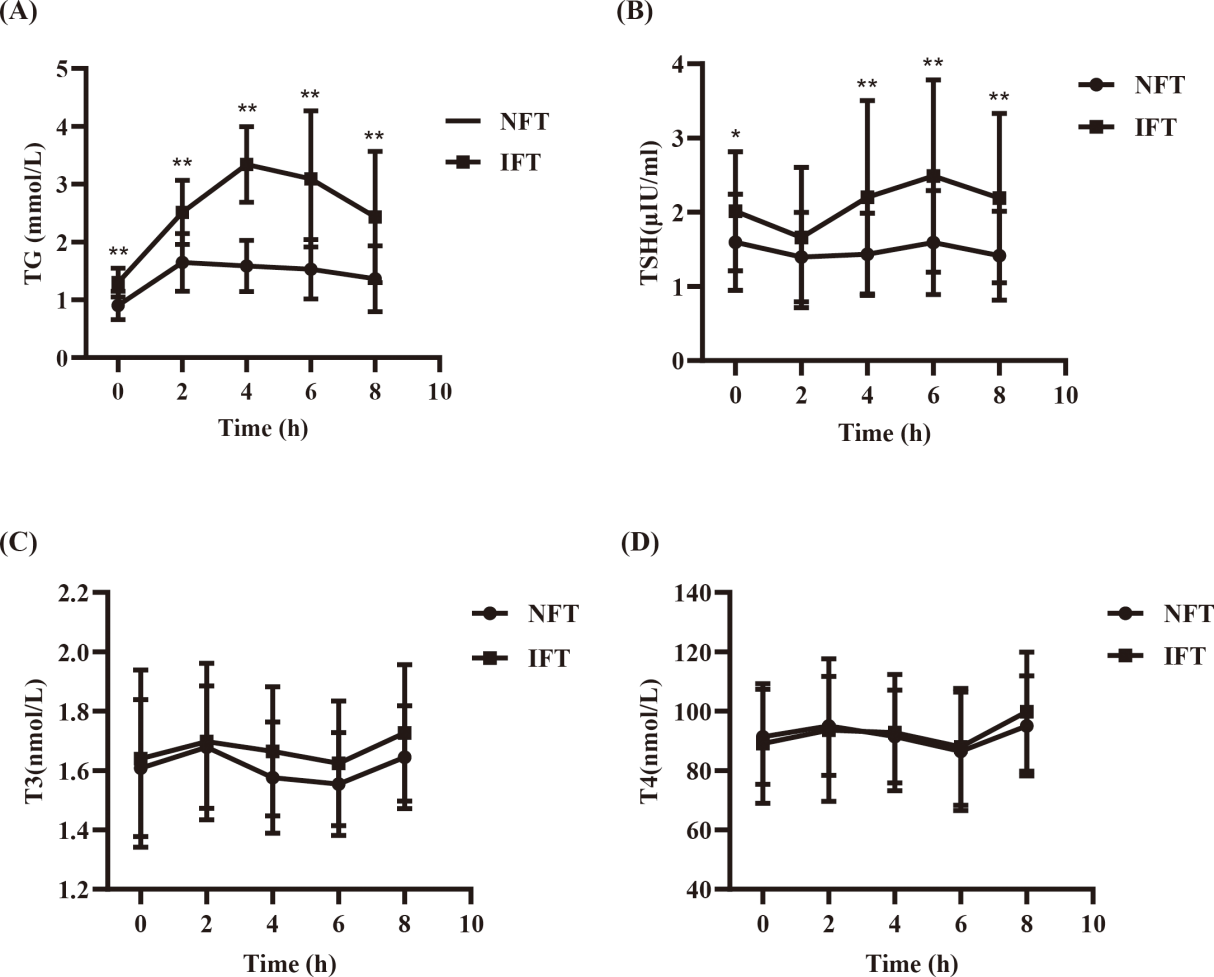
**(A)** Comparison of the Serum TG levels between two groups during the OFTT. **(B)** Comparison of the SerumTSH levels between two groups during the OFTT. **(C)** Comparison of the Serum T3 levels between two groups during the OFTT. **(D)** Comparison of the Serum T4 levels between two groups during the OFTT. *P<0.05, compared with the NFT group at different time points. **P<0.001, compared with the NFT group at different time points.

Postprandial TG levels in the NFT and IFT groups increased over time. The NFT group reached peak TG levels at 2 hours postprandially, while the IFT group reached peak TG levels at 4 hours postprandially. There were statistically significant differences in TG levels between the two groups at all time points (P < 0.001). The TGAUC were also significantly different between the two groups (P < 0.001). Repeated-measures analysis of variance showed that the changes in postprandial TG levels over time were statistically different between the two groups (P < 0.001), and there were statistically significant differences between the two groups (P < 0.05). △TG and TGAUC in the IFT group were higher than those in the NFT group, with statistical significance (P < 0.001). Individuals with reduced fat tolerance exhibit a delayed peak time and higher peak values of postprandial TG after consuming a high-fat meal than those with normal fat tolerance (**Table 2**, **Figure 1A**).

**Table 2:** Comparison of the Serum TG levels between two groups during the OFTT.

TG (mmol/L)	0h	2h	4h	6h	8h	△TG	TGAUC
NFT	0.90 ± 0.25	1.64 ± 0.50	1.59 ± 0.45	1.53 ± 0.51	1.36 ± 0.57	0.74 ± 0.44	11.78 ± 2.86
IFT	1.30 ± 0.25 **	2.51 ± 0.56 **	3.34 ± 0.66 **	3.09 ± 1.18 **	2.43 ± 1.14 **	2.04 ± 0.66**	21.60 ± 4.59 **

*P<0.05, compared with the NFT group at different time points, **P<0.001, compared with the NFT group at different time points.

### Comparison of the Serum TSH levels between two groups during the OFTT

Postprandial TSH levels in both NFT and IFT groups initially decreased, reaching a nadir at 2 hours, and then increased, peaking at 6 hours, with the IFT group showing a higher peak. Compared with the NFT group, except for the 2-hour mark, there were statistically significant differences in TSH levels at all other time points in the IFT group (P < 0.05). △TSH and TSHAUC in the IFT group were higher than those in the NFT group, with statistical significance (P < 0.001). Repeated-measures analysis of variance indicated that the changes in postprandial TSH levels over time were statistically different between the two groups (P < 0.05), and there were statistically significant differences between the two groups (P < 0.05) (**Table 3**, **Figure 1B**).

**Table 3:** Comparison of the Serum TSH levels between two groups during the OFTT.

TSH (uIU/mL)	0h	2h	4h	6h	8h	△TSH	TSHAUC
NFT	1.52 (1.00,2.02)	1.31 (1.00,1.72)	1.39 (1.04,1.82)	1.36 (1.10,2.07)	1.37 (1.00,1.68)	-0.02 (-0.30,-0.21)	10.84 (8.36,14.39)
IFT	1.82 (1.38,2.34)*	1.36 (1.05,2.08)	1.85 (1.51,2.30)**	2.18 (1.72,2.93)**	1.94 (1.47,2.52)**	0.38 (0.12,0.85) **	16.25 (11.79,20.31)**

*P<0.05, compared with the NFT group at different time points, **P<0.001, compared with the NFT group at different time points.

### Comparison of the Serum T3 and T4 levels between two groups during the OFTT

Postprandial T3 and T4 levels in both NFT and IFT groups initially increased, peaked at 2 hours, and then decreased, reaching a nadir at 6 hours. Repeated-measures analysis of variance showed that the changes in postprandial T3 and T4 over time were statistically different all the two groups (P < 0.05); however, there were no statistically significant differences in the comparison between two groups. There were also no differences in triiodothyronine area under the curve (T3AUC) and thyroxine area under the curve (T4AUC) between the two groups (P > 0.05) (**Tables 4**, **5**, **Figures 1C, D**).

**Table 4:** Comparison of the Serum T3 levels between two groups during the OFTT.

T3 (nmol/L)	0h	2h	4h	6h	8h
NFT	1.61 ± 0.23	1.68 ± 0.21	1.58 ± 0.19	1.55 ± 0.17	1.65 ± 0.17
IFT	1.64 ± 0.30	1.70 ± 0.26	1.67 ± 0.22	1.62 ± 0.21	1.73 ± 0.23

**Table 5:** Comparison of the SerumT4 levels between two groups during the OFTT.

T4 (nmol/L)	0h	2h	4h	6h	8h
NFT	91.35 ± 16.00	95.02 ± 16.64	91.47 ± 15.63	86.50 ± 20.01	95.07 ± 16.93
IFT	89.09 ± 20.20	93.59 ± 24.03	92.79 ± 19.55	87.99 ± 19.70	99.85 ± 20.10

### Relationship between TGAUC and TSHAUC

There was a positive correlation between TGAUC and TSHAUC in both groups (r = 0.386, P < 0.05). Univariate linear regression analysis showed that TGAUC was a influencing factor for TSHAUC (P < 0.01). After adjustment for age, sex, BMI, SBP, DBP, FBG and TC, multiple linear regression analysis showed that TGAUC was an independent influencing factor for TSHAUC(P < 0.001).

## Discussion

With improvements in living standards, the prevalence of hyperlipidemia keeps increasing. Clinically, the lipid metabolism state of the human body is often determined by the fasting blood lipid level. However, during most of the day, the body is in the postprandial state, so the detection of fasting blood lipids does not reflect the overall picture of lipid metabolism. In recent years, many studies have shown that postprandial TG levels are closely associated with type 2 diabetes and cardiovascular disease (13–15). An increasing number of researchers are focused on the importance of postprandial TG. Based on the importance of postprandial TG, we used the oral fasting tolerance test to observe the changes in postprandial TG levels over time in participants with normal fasting TG levels after eating a high-fat meal to observe the overall lipid metabolism status.

The first important finding of our study was that TG levels were increased postprandially in participants in both groups. Compared with the NFT group, even though the fasting TG level in the ITF group was normal, the postprandial TG peak was higher and delayed. The △ TG and TG AUCs were also significantly higher than those of the NFT group. These findings suggest that a reduced lipid tolerance is hidden in individuals with normal fasting TG levels, which is an early stage of lipid metabolism disorder. Therefore, in our clinical work, attention should be paid to identifying patients with lipid metabolism disorders in the early stage, which has important guiding significance for the prevention and management of hyperlipidemia and its complications.

Previous clinical studies have shown that fasting TSH levels are also elevated in individuals with a high fasting TG-emia, compared with those with normal fasting TG levels (10–12). However, all previous studies were based on fasting TG levels, and no study has investigated the effect of postprandial TG on postprandial TSH levels. Based on the significance of postprandial TG, we observed the relationship between postprandial TG levels and postprandial TSH levels in individuals with normal fasting TG levels.

The second important finding of our study was that TGAUC was an independent influencing factor of TSHAUC and was positively correlated. There were statistically significant differences in BMI, SBP, DBP, and FBG between the two groups, and abnormalities in these indicators are often present in people with lipid metabolism disorders, which may be potential factors affecting TSH secretion. Therefore, after we further corrected for these potential confounders, we found that TGAUC remained an independent influencing factor for TSHAUC. This suggests that the postprandial TSH levels are influenced by the postprandial TG levels.

Some studies on organ damage caused by lipotoxicity have confirmed that excessive dietary fat intake triggers ectopic lipid deposition, leading to cellular function damage, also known as “lipotoxic damage.” This interferes with the endocrine system and leads to the development of diseases, such as thyroid disease, especially hypothyroidism (21–23). Shao Shanshan et al. (24) found that when rats consumed a diet containing high-fat lard for some time, serum TSH level was significantly increased, serum T4 and FT4 levels were significantly decreased, and serum T4 level was negatively correlated with serum TG level. The team also found that the TG content in the thyroid was significantly increased, the morphology and ultrastructure of the thyroid were changed, and the protein expression level related to thyroid hormone synthesis decreased. These findings suggest that lipotoxicity may be involved in the development of thyroid dysfunction. MinHee Lee et al. (25) found that all three mice showed varying degrees of primary hypothyroidism after receiving a high-fat diet for some time. Lipid deposition and ultrastructural changes, including endoplasmic reticulum swelling and mitochondrial deformation of thyroid cells, were observed in thyroid tissue. These findings indicated that high-fat diet-induced lipotoxicity damages thyroid tissue and participates in the development of thyroid dysfunction. In a mechanistic study of high-fat diet-induced hypothyroidism in rats by Wang et al. (26), it was found that endoplasmic reticulum stress occurred in rat thyroid cells, which reduced the expression level of thyroglobulin, a key molecule of thyroid hormone synthesis. The research team also produced the same results using a palmitate-induced high-fat model of human primary thyrocytes. This evidence indicates that the high-fat diet-induced lipotoxicity can change the thyroid morphology, increase the endoplasmic reticulum stress, and promote a decline in thyroid function. These findings strongly support our results, and we further identified that the postprandial TG levels affect the postprandial TSH level based on previous studies. This study fills the gap in the effect of postprandial TG on postprandial TSH levels and further explores the effects of lipid metabolism disorders on thyroid function.

The third important finding of our study was that the postprandial TSH levels in both groups reached a trough at 2 h and peaked at 6 h, with the opposite trend of T3 and T4 levels and TSH. All the postprandial TSH levels reached a trough at 2 h, which is consistent with the findings of previous studies. TSH secretion primarily depends on two factors: thyrotropin-releasing hormone and somatostatin; the former stimulates TSH secretion, while the latter inhibits it (27). A study by Ehrenkranz et al. based on large-scale laboratory data showed significant diurnal rhythmic changes in circulating TSH levels. Despite the pulsatile secretion of TSH, the low amplitude of pulses and long half-life of TSH result in only minor fluctuations in blood TSH levels (28). One possible reason for the acute decline in TSH postprandially is the suppression of TSH secretion due to an increase in circulating somatostatin levels induced by food (29). After consuming a high-fat meal, the levels of lipoproteins rich in TG, such as chylomicrons and very-low-density lipoproteins, increase in the body; thyroid hormones can hydrolyze TG-rich lipoproteins by regulating the activity of lipoprotein lipase, thereby reducing circulating TG levels (30). Thyroid hormones also participate in the regulation of the rate-limiting enzyme CPT-1α in fatty acid β-oxidation by inducing the activation of Akt, thus reducing circulating levels of fatty acids and TG (31). Therefore, after a high-fat meal is consumed, the increase in TG-rich lipoproteins in the body prompts the thyroid to respond to the next step of fat metabolism, with the secretion of T3 and T4 increasing 2 h postprandially. This leads to a transient decrease in TSH levels through negative feedback regulation, which may be another important reason for the decrease in postprandial TSH levels. Surprisingly, we found that TSH levels peaked at 6 h after the meal, and T3 and T4 reached their lowest values. TGAUC is an independent influencing factor of TSHAUC and is positively correlated, which indicates that the increase in postprandial TSH levels is influenced by increased postprandial TG levels. We speculate that after a high-fat meal, the circulating TG levels increase, and the demand for thyroid hormones involved in fat metabolism increases. Consequently, the consumption of T3 and T4 increases, causing their levels to reach their lowest values. This triggers negative feedback regulation, leading to increased TSH secretion, which explains the observed peak.

The fourth and most important finding of our study was that the postprandial peak TSH, △ TSH, and TSHAUC were significantly higher in the IFT group than in the NFT group. This indicates that participants in the IFT group had a trend towards hypothyroidism. Based on the conclusions of previous studies on the mechanism of lipotoxic damage to thyroid function and the conclusion of this study that postprandial TG levels affect postprandial TSH levels, it can be speculated that TG clearance is slowed down after high-fat meals are consumed. This continuous postprandial high TG state can aggravate the thyroid damage caused by lipotoxicity and lead to hypothyroidism. This suggests that we should not only focus on thyroid function in people with elevated fasting TG levels but also on thyroid function in people with early lipid metabolism disorders with normal fasting TG levels but elevated postprandial TG levels. The findings of our study provide an important clinical basis for the effective prevention and management of SCH.

With the improvement in living standards, the prevalence of hyperlipidemia is increasing. Previous studies have emphasized that people with elevated fasting TG are prone to hypothyroidism. Based on previous studies, we further found that in individuals with normal fasting TG levels, the hidden individuals with elevated postprandial TG levels are those with early lipid metabolism disorders, and their fasting and postprandial TSH levels are also higher than those of individuals with normal postprandial TG levels, who are prone to hypothyroidism. This prompts us to pay attention to identifying people with early lipid metabolism disorders and their thyroid function. This has important clinical implications for early detection and better management of lipid metabolism disorders and hypothyroidism.

Some limitations of this study need to be addressed. First, this study is our initial exploration of the effect of postprandial TG levels on postprandial thyroid function. Due to our limited experimental conditions and the sample size not being large enough, we will expand the sample size in the future to further validate this study’s results. Second, there is no mechanistic study carried out in this study. The next step will consider animal and cell experiments for an in-depth analysis of the interaction relationship and the potential mechanisms.

In conclusion, our study shows that after a high-fat meal is consumed, postprandial TSH levels are influenced by postprandial TG levels. The decrease in lipid tolerance not only decreases TG clearance ability but also decreases thyroid function. This is the first study in which an oral fasting tolerance test was used to identify higher fat postprandial TG levels and higher postprandial TSH levels in people with early lipid metabolism disorders than in those with normal lipid metabolism. Our findings have important clinical implications for the effective prevention and management of lipid metabolism disorders and hypothyroidism.

## Data Availability

The original contributions presented in the study are included in the article/supplementary material. Further inquiries can be directed to the corresponding author.
